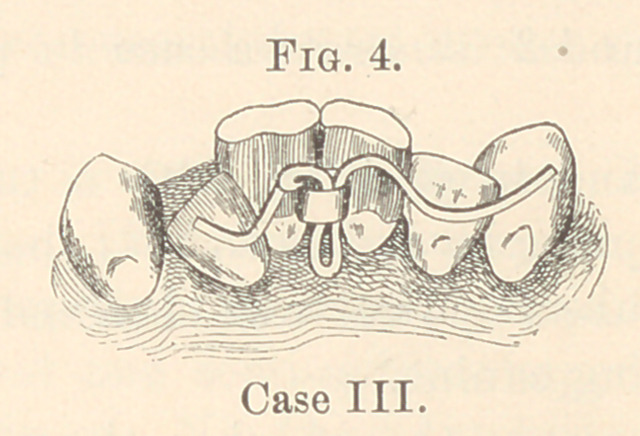# Spring Levers for Regulating Teeth

**Published:** 1898-01

**Authors:** W. S. Davenport

**Affiliations:** Paris, France


### SPRING LEVERS FOR REGULATING TEETH.[1]

BY W. S. DAVENPORT, PARIS, FRANCE.

Case I.—Figs. 1 and 2 illustrate a case in practice before and after treatment.

Fig. 2 shows a band in position, with a tube soldered to one side. A piano-wire spring lever was so bent that when one end was passed through the tube *P*, power was obtained by pressing tightly against the neighboring canine.

The tube *W* represents the weight; the point *F* becomes the fulcrum by touching the outer edge of the tooth to be rotated. With this simple appliance a tooth becomes practically self-rotating.

---

[1] Presented to the American Dental Club, of Paris.

Case II.—A cap was made with a post soldered to one corner; the lever was slipped over the post and the end sprung up against

Fig. 1.

Fig. 2.

Case I.

the neck of the molar, giving a force as obtained in a lever of the second class. See *P*, *W*, and *F*.

Fig. 3.

Case II.

Case III.—A double gold band with a flattened tube attached was cemented to the two central incisors. Through the tube was

Fig. 4.

Case III.

sprung the double spring lever, which brought force to bear on the two laterals, the position of which was thus corrected.

Every day or two the springs were removed and bent a little more, which gave renewed power to continue their action.

## Figures and Tables

**Case I. f1:**
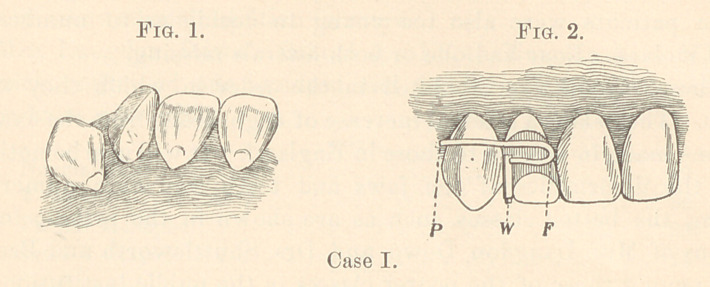


**Case II. f2:**
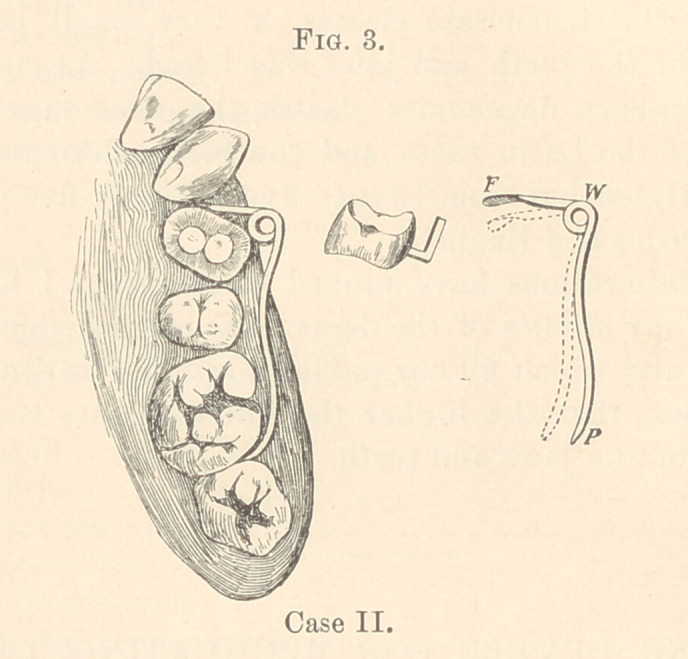


**Case III. f3:**